# OsJAC1 Positively Regulates Brown Planthopper Resistance in Rice Through Priming of Diterpenoid Biosynthesis and Amplification of Jasmonic Acid Signaling

**DOI:** 10.3390/plants15182840

**Published:** 2026-09-17

**Authors:** Jie Wang, Na Sun, Runbin Zhong, Jinyi Tian, Rongrong Xue, Yingying Zheng, Yuanyuan Song, Rensen Zeng, Dongmei Chen

**Affiliations:** 1 Key Laboratory of Ministry of Education for Genetics, Breeding and Multiple Utilization of Crops, State Key Laboratory of Agricultural and Forestry Biosecurity, College of Agriculture, Fujian Agriculture and Forestry University, Fuzhou 350002, China; jw0813@fafu.edu.cn (J.W.);; 2 Key Laboratory of Crop Biotechnology of Fujian Higher Education Institutes, Key Laboratory of Biological Breeding for Fujian and Taiwan Crops, Ministry of Agriculture and Rural Affairs, Fujian Agriculture and Forestry University, Fuzhou 350002, China; 3 Key Laboratory of Vector Biology and Pathogen Control of Zhejiang Province, School of Life Sciences, Huzhou Normal University, Huzhou 330500, China

**Keywords:** *Oryza sativa*, *Nilaparvata lugens*, *OsJAC1*, jasmonic acid, diterpenoid, plant-insect interaction

## Abstract

Brown planthopper (BPH) is a devastating rice pest worldwide, and enhancing intrinsic plant resistance represents a sustainable strategy for its control. Here, we report that *OsJAC1*, a gene encoding a protein harboring dirigent and jacalin-related lectin domains, positively regulates BPH resistance in rice by priming diterpenoid biosynthesis and amplifying jasmonic acid (JA) signaling. Overexpression of *OsJAC1* (OE) in *japonica* cv. Zhonghua 11 significantly enhanced resistance, whereas CRISPR/Cas9 knockout (KO) increased susceptibility. Transcriptomic profiling revealed that OE plants constitutively upregulated diterpenoid biosynthetic genes, including *OsCPS4*, *OsKSL4* and *CYP99A3*, with further induction upon BPH infestation, accompanied by enrichment of MYB and bHLH transcription factors. Targeted metabolomics showed elevated momilactone A and B levels in OE plants. Hormonal assays demonstrated that JA accumulation was amplified in OE after BPH attack. Collectively, OsJAC1 confers resistance via a dual mechanism: constitutive priming of diterpenoid metabolism and attack-triggered amplification through JA signaling, but the precise biochemical function remains to be determined. Our findings uncover a regulatory cascade from transcriptional activation to phytoalexin accumulation and identify *OsJAC1* as a promising target for breeding BPH-resistant rice.

## 1. Introduction

Rice (*Oryza sativa* L.) is a staple food for more than half of the global population, and brown planthopper (BPH, *Nilaparvata lugens* Stål) is one of the most destructive insect pests of rice, causing substantial annual yield losses through direct feeding and the transmission of viral diseases [1]. BPH is a phloem-feeding insect that predominantly infests the leaf blades and leaf sheaths of rice plants [2]. The development of resistant rice varieties represents the most economical and environmentally sustainable strategy for BPH management [3].

The OsJAC1 protein contains an N-terminal dirigent (DIR) domain and a C-terminal jacalin-related lectin (JRL) domain [4]. DIR domains are involved in stereoselective coupling of secondary metabolites, particularly in lignin biosynthesis [5], while JRL domains function as lectins that recognize carbohydrate signals [6], and recent studies have further highlighted the role of JRL proteins in plant defense [7]. Plant lectins, especially JRLs, have been extensively studied for their direct insecticidal activities [8,9]. These findings have established a classic paradigm that JRL proteins act as “protein insecticides” that directly kill insects by binding to gut glycoproteins. Previous studies have demonstrated that *OsJAC1*, whose promoter harbours jasmonic acid (JA)-responsive cis elements, and *OsJAC1*-overexpressing rice plants display enhanced sensitivity to exogenous JA accompanied by suppressed coleoptile elongation [10]. *OsJAC1* overexpression has been demonstrated to confer broad-spectrum resistance against bacterial, oomycete, and fungal pathogens in rice and barley, and the JRL domain of OsJAC1 alone can localize to pathogen infection sites [4]. Interestingly, a recent study generated *osjac1* knockout mutants in the japonica variety Zhonghua 11 and found that these mutants exhibit enhanced resistance to rice blast and bacterial blight, indicating that OsJAC1 acts as a negative regulator of disease resistance in certain contexts, functioning as a susceptibility gene [11].

The phytohormone JA plays a central role in plant defense against herbivorous insects [12,13,14]. In rice, JA signaling is crucial for BPH resistance [15,16], and Jiang et al. (2007) reported that *OsJAC1* was JA- inducible [10]. However, the function of OsJAC1 in defense against insect herbivores, particularly BPH, has not been explored. In this study, we investigated the role of OsJAC1 in rice–BPH interaction using *OsJAC1*-overexpressing lines and CRISPR/Cas9-generated knockout mutants in the ZH11 background. We show that OsJAC1 positively regulates BPH resistance by priming diterpenoid biosynthesis and amplifying JA signaling.

## 2. Results

### 2.1. OsJAC1 Is Predominantly Expressed in Leaves and Leaf Sheaths and Rapidly Induced by BPH and MeJA

**Figure 1:**
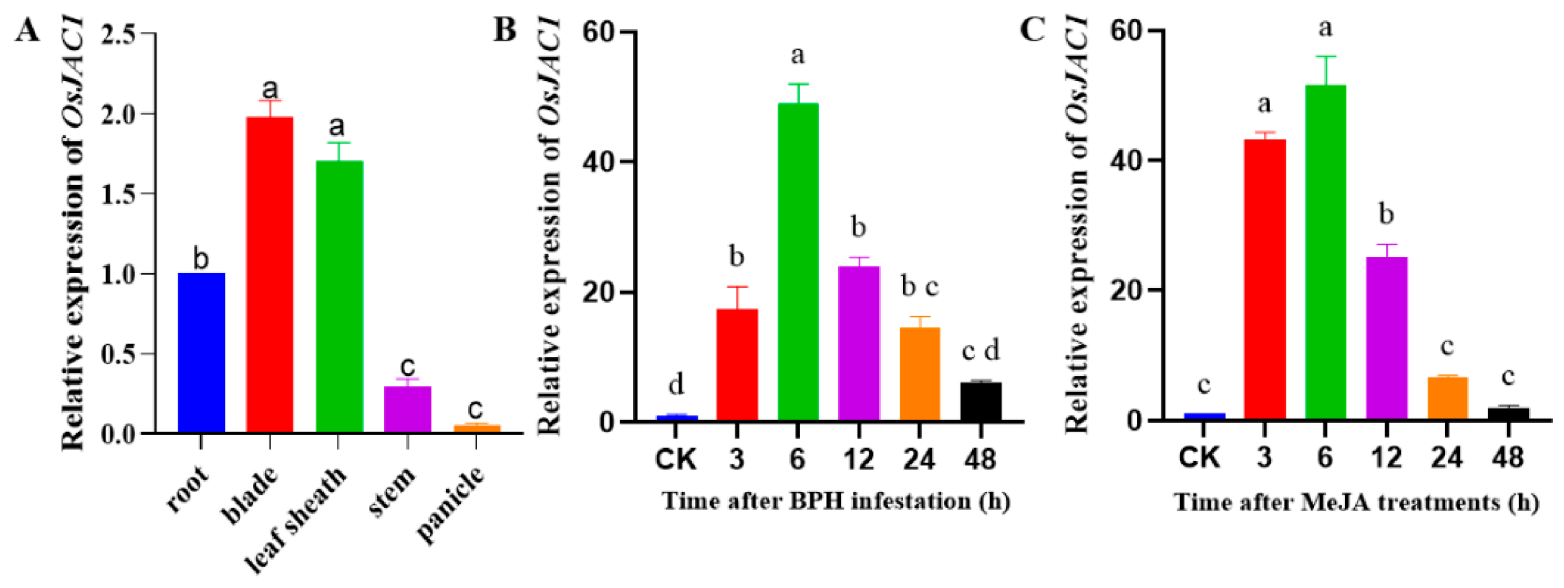
Expression pattern and induction dynamics of *OsJAC1*. (**A**) Tissue-specific expression of *OsJAC1* in root, blade, leaf sheath, stem and panicle determined by qPCR. (**B**) Time-course expression of *OsJAC1* after BPH infestation. (**C**) Time-course expression of *OsJAC1* after 100 µM MeJA treatment. Data are mean ± SE (n = 3), and different letters indicate significant differences among treatments (*p* < 0.05, one-way ANOVA followed by Tukey‘s HSD test).

To characterize the expression pattern of *OsJAC1*, we first examined its transcript levels in various rice tissues. qPCR analysis revealed that *OsJAC1* was predominantly expressed in leaf blades and leaf sheaths, with lower expression in roots, stems and panicles (Figure 1A). We then examined its temporal expression dynamics upon BPH infestation. *OsJAC1* transcripts accumulated rapidly, reaching peak levels at 6 h post-infestation (Figure 1B). To determine whether *OsJAC1* induction is mediated by JA signaling, we treated seedlings with 100 µM MeJA, which induced a similar temporal pattern with peak expression also at 6 h (Figure 1C). The preferential expression in BPH feeding sites, together with the rapid and synchronized induction of *OsJAC1*, suggests that OsJAC1 acts as an early component of the JA signaling pathway under BPH infestation.

### 2.2. OsJAC1 Positively Regulates BPH Resistance

**Figure 2:**
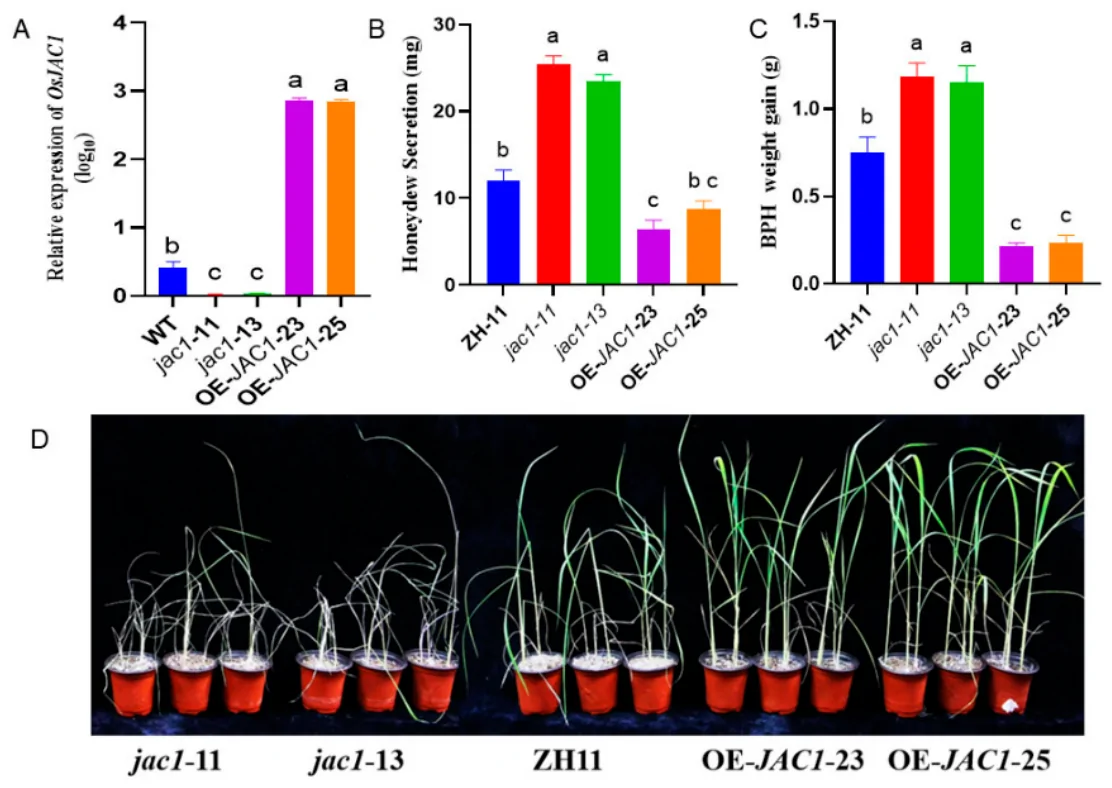
*OsJAC1* positively regulates BPH resistance. (**A**) Relative expression of *OsJAC1* in WT, OE lines (OE-*JAC1*-23 and OE-*JAC1*-25), and KO lines (*osjac1*-11 and *osjac1*-13). The y-axis is shown on a logarithmic scale (log10). (**B**) Honeydew excretion (mg) of BPH nymphs after 48 h of feeding on WT, OE and KO plants. (**C**) Body weight gain (mg) of BPH nymphs after 48 h of feeding on the respective genotypes. (**D**) Representative phenotypes of WT, OE and KO plants at 7 days after BPH infestation. Data are mean ± SE (n = 3 for qPCR; n = 15–20 insects per genotype for honeydew and weight gain), and different letters indicate significant differences between treatments (*p* < 0.05, one-way ANOVA followed by Tukey’s HSD test).

To investigate the function of OsJAC1 in BPH resistance, we generated *OsJAC1*-overexpressing lines (OE-*JAC1*-23 and OE-*JAC1*-25) via Agrobacterium-mediated transformation and knockout mutants (*osjac1*-11 and *osjac1*-13) via CRISPR/Cas9-mediated gene editing, both in the ZH11 background. The mutations in the KO lines resulted in frameshifts and premature stop codons, predicting truncated non-functional proteins. qPCR analysis confirmed that *OsJAC1* expression was dramatically increased in OE lines (several hundred-fold higher than WT) and was undetectable in KO mutants (Figure 2A). Under normal growth conditions, no obvious morphological differences were observed among WT, OE and KO plants, except that OE plants were slightly shorter (Supplementary Figure S1). However, field yield data were not collected in this study, and the effect on grain yield under field conditions remains to be evaluated. BPH feeding assays showed that honeydew excretion was significantly reduced on OE plants compared to WT, while KO plants exhibited increased honeydew production (Figure 2B). Consistently, BPH nymphs feeding on OE plants gained significantly less weight than those on WT, whereas those on KO plants gained more weight (Figure 2C). At 7 days after infestation, OE plants remained healthy and green, while KO plants showed severe wilting and chlorosis (Figure 2D). These results demonstrate that OsJAC1 functions as a positive regulator of BPH resistance in rice.

### 2.3. Transcriptomic Analysis Reveals Constitutive Activation and Further Amplification of Diterpenoid Biosynthesis by OsJAC1

**Figure 3:**
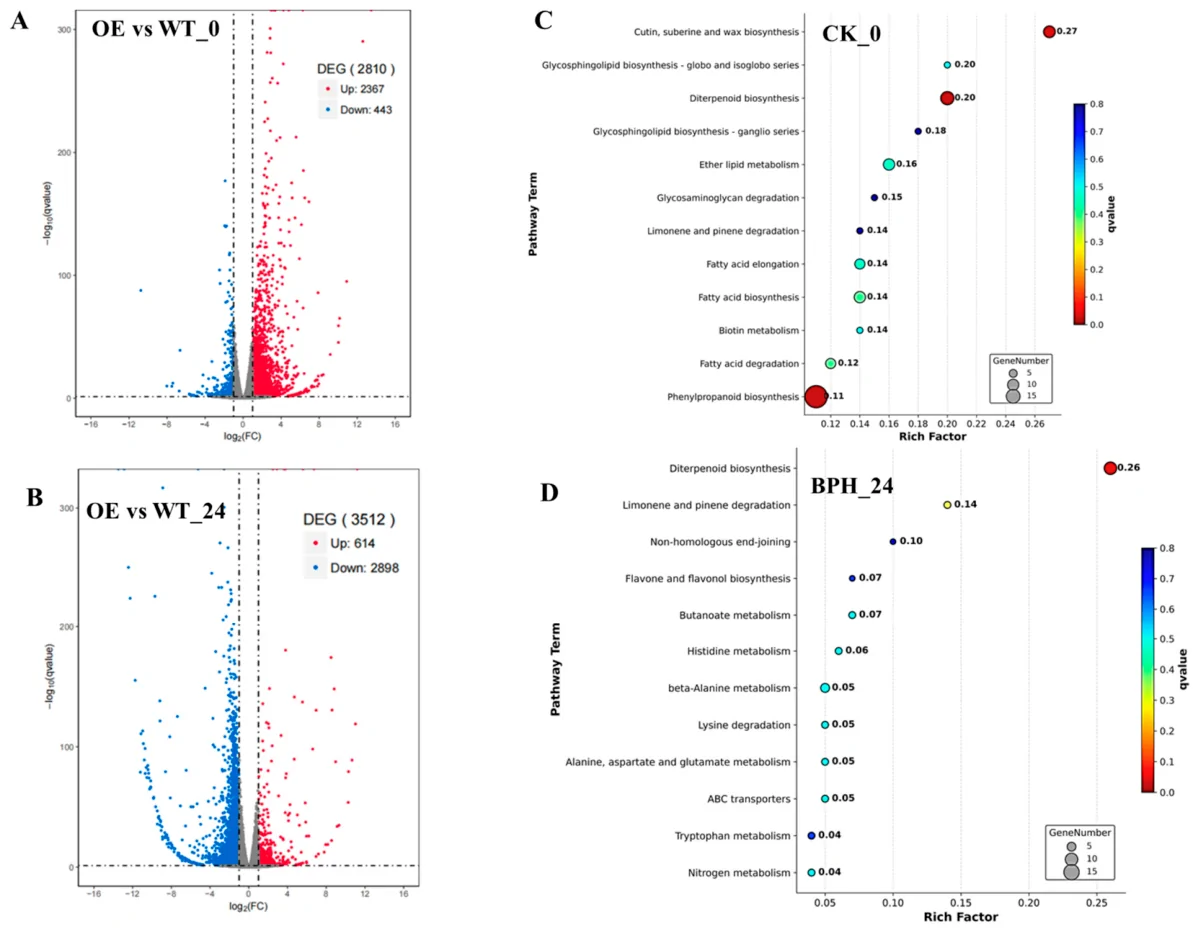
Transcriptomic analysis reveals diterpenoid biosynthesis as the key pathway enriched by *OsJAC1*. Volcano plots of differentially expressed genes in OE vs. WT at 0 h (**A**) and differentially expressed genes in OE vs. WT at 24 h (**B**). Red and blue dots represent upregulated and downregulated genes, respectively (log_2_FC ≥ 1, *p* < 0.05). (**C**) Enrichment analysis of the top 12 KEGG pathways of upregulated genes in OE vs. WT at 0 h (**C**) and 24 h (**D**). Note that diterpenoid biosynthesis and phenylpropanoid biosynthesis pathways were significantly enriched at both time points. The Rich Factor represents the enrichment degree of the pathway. The bubble size indicates the number of differentially expressed genes within the pathway, and the bubble colour represents the *p-*value. A redder colour indicates higher enrichment significance.

To understand the molecular basis of OsJAC1-mediated defense, we performed transcriptome sequencing on WT and the OE-23 line at 0 and 24 h after BPH infestation. We selected 0 h to capture constitutive differences (primed state) and 24 h to capture the established defense response. Principal component analysis (PCA) showed that WT and OE plants at 0 h without BPH infestation significantly separated from each other, while WT and OE plants at 24 h after BPH infestation clustered together with a slight separation (Figure S2). We focused our analysis on the constitutive differences at 0 h and 24 h, which provided the molecular basis for the primed defense state and induced defense state in OE plants, respectively. Differential expression analysis at 0 h identified 2387 upregulated and 443 downregulated genes in OE compared to WT (Figure 3A). At 24 h, 614 genes were upregulated and 2898 were downregulated in OE compared to WT, as shown in volcano plots (Figure 3B). The striking reversal in DEG numbers—from a broad constitutive upregulation at 0 h to a more focused response at 24 h—suggests that OE plants are constitutively primed, and upon BPH attack they prioritize specific defense pathways while downregulating general stress responses, whereas WT plants mount a broad, unprimed stress response. The details of all the differentially expressed genes (DEGs) were demonstrated in Table S2. KEGG enrichment analysis of the upregulated genes revealed that diterpenoid biosynthesis was significantly enriched at both 0 h and 24 h, whereas phenylpropanoid biosynthesis was significantly enriched only at 0 h (Figure 3C,D; Table 1). Notably, while the total number of upregulated genes decreased over time, the enrichment of diterpenoid biosynthesis pathway remained prominent, indicating a focused transcriptional response on this specific defense pathway.

### 2.4. Diterpenoid Biosynthesis Pathway Is Transcriptionally and Metabolically Activated by OsJAC1

**Figure 4:**
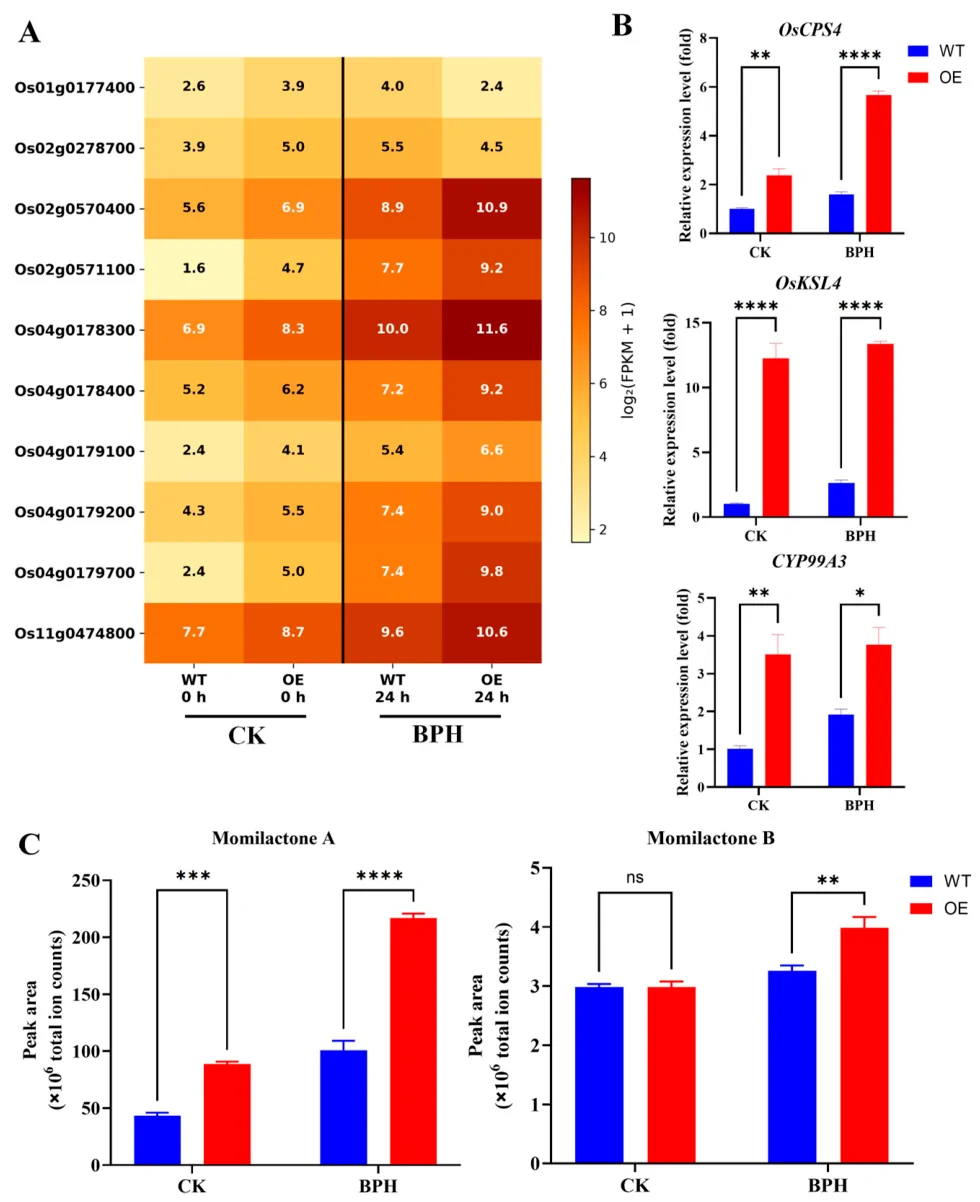
The diterpenoid biosynthesis pathway is transcriptionally and metabolically activated by *OsJAC1*. (**A**) Heatmap showing expression of genes involved in the diterpenoid biosynthesis pathway in WT and OE at 0 h and 24 h after BPH infestation. (**B**) qPCR validation of *OsCPS4*, *OsKSL4*, and *CYP99A3* in WT and OE at 0 h and 24 h after BPH infestation. (**C**) Momilactone A and momilactone B contents in WT and OE at 24 h after BPH infestation. Data are mean ± SE (n = 3). * *p* < 0.05, ** *p* < 0.01, *** *p* < 0.001, **** *p* < 0.0001; ns, not significant.

Based on the transcriptomic results, we focused on the diterpenoid biosynthesis pathway for further validation. Heatmap analysis of genes involved in momilactone biosynthesis showed that ten key biosynthetic genes, including *OsCPS4*, *OsKSL4*, and *CYP99A3*, were consistently upregulated in OE plants compared to WT at both 0 h and 24 h (Figure 4A). qPCR analysis confirmed the upregulation of *OsCPS4*, *OsKSL4*, and *CYP99A3* in OE plants at both time points (Figure 4B). To determine whether the transcriptional activation leads to accumulation of corresponding metabolites, we measured the levels of momilactone A and momilactone B at 24 h after BPH infestation. OE plants accumulated significantly higher levels of both momilactone A and momilactone B compared to WT (Figure 4C). These results confirm that *OsJAC1* overexpression activates the diterpenoid biosynthesis pathway at both transcriptional and metabolic levels.

### 2.5. OsJAC1 Enhances JA Accumulation and Activates Transcription Factors

**Figure 5:**
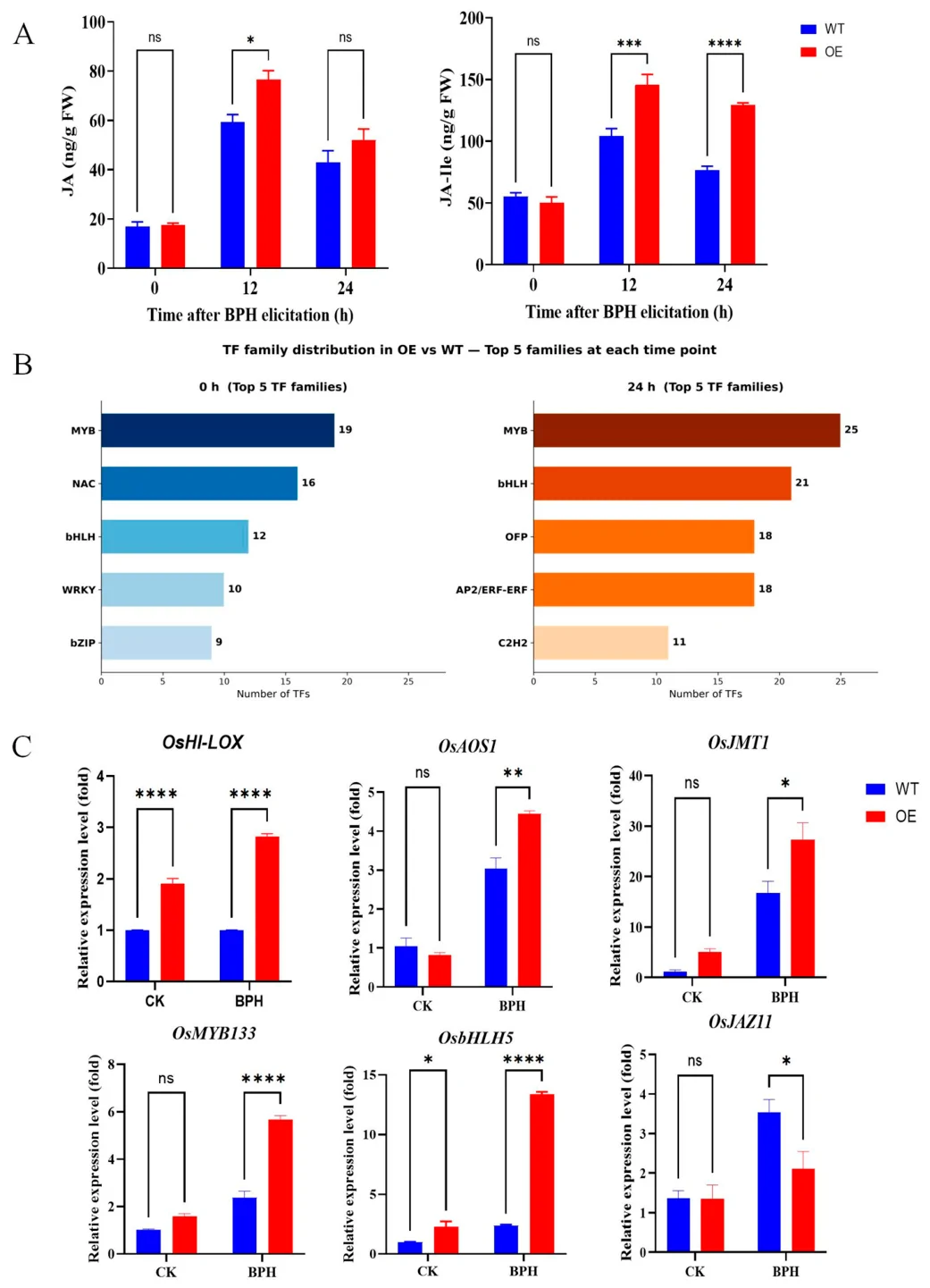
*OsJAC1* enhances JA accumulation and activates transcription factors. (**A**) JA & JA-Ile content in WT and OE at 0, 12 and 24 h post-infestation. (**B**) Top 5 transcription factor families enriched among upregulated genes in OE vs. WT at 0 h and 24 h. (**C**) Expression of JA signaling-related and regulated transcription factor genes in WT and OE at 0 h (CK) and 24 h after BPH infestation (BPH). Data are mean ± SE (n = 3). * *p* < 0.05, ** *p* < 0.01, *** *p* < 0.001, **** *p* < 0.0001; ns, not significant.

In transcriptomic analysis, the most significantly enriched transcription factor families among upregulated genes in OE vs. WT at both 0 h and 24 h after BPH infestation were MYB and bHLH (Figure 5C), which are known to mediate plant secondary metabolism and be regulated by JA signaling. To elucidate the mechanistic basis for the enhanced transcriptional and metabolic response, we measured JA and JA-Ile levels in WT and OE plants at different time points after BPH infestation. Under uninfested conditions (0 h), no significant differences in JA and JA-Ile content were observed (Figure 5A). Upon BPH infestation, OE plants exhibited a robust JA and JA-Ile accumulation, with both JA and JA-Ile levels significantly higher than WT at 12 h (Figure 5A). To further confirm the activation of JA signaling in OE plants, we examined the expression of selected JA-related genes and transcription factors by qPCR. The positive regulators, including *OsHI-LOX*, *OsAOS1*, *OsJMT1*, *OsMYB133* and *OsbHLH5* genes, were significantly upregulated in OE plants, while the negative regulator *OsJAZ11* was downregulated (Figure 5C). These data indicate that *OsJAC1* amplifies JA accumulation upon BPH attack and activates downstream transcription factors, providing a mechanistic explanation for the enhanced expression of diterpenoid biosynthetic genes in OE plants.

## 3. Discussion

In the present study, we combined transcriptomic, metabolomic, hormonal, and genetic analyses to elucidate the mechanistic basis of OsJAC1-mediated resistance against BPH in rice plants. Our results demonstrate that OsJAC1 functions as a positive regulator of BPH resistance by amplifying JA signaling, which in turn drives the transcriptional activation of transcription factors from MYB and bHLH families, ultimately promoting the accumulation of diterpenoid phytoalexins. This regulatory cascade establishes a primed defense state in *OsJAC1*-overexpressing plants, enabling a rapid and robust response upon pest attack without incurring constitutive growth penalties.

Hormonal analysis revealed that BPH infestation triggered a significantly stronger JA burst in *OsJAC1*-OE plants compared to WT controls. This amplification of JA signaling provides a mechanistic explanation for the enhanced transcriptional reprogramming observed in OE plants. JA is a well-established central regulator of diterpenoid biosynthesis in rice [17], and our transcriptomic data showed that the diterpenoid biosynthetic pathway was consistently enriched in OE plants at both 0 h and 24 h after infestation. Notably, key biosynthetic genes including *OsCPS4*, *OsKSL4*, and *CYP99A3*, which encode the core enzymes for momilactone biosynthesis, were significantly upregulated in a JA-dependent manner [18,19,20]. Targeted metabolomic analysis further confirmed that momilactone accumulated to substantially higher levels in OE plants, establishing a complete regulatory cascade from JA amplification to transcriptional activation and ultimately to diterpenoid phytoalexin accumulation. Future time-course transcriptomic and hormonal analyses at earlier time points will be valuable to fully resolve the primary and secondary waves of this defense response.

The observed downregulation of *OsJAZ11* in *OsJAC1*-OE plants provides additional molecular evidence for enhanced JA signaling. JAZ proteins function as key repressors of JA responses by interacting with and inhibiting downstream transcription factors; upon JA perception, they are targeted for degradation via the 26S proteasome pathway, thereby releasing the repression of JA-responsive genes [21]. The reduced *OsJAZ11* transcript level in OE lines is likely consistent with an amplified JA burst, which likely accelerates JAZ11 turnover and attenuates its negative regulation of the JA pathway [22]. Concurrently, the coordinated upregulation of *OsMYB133* and *OsbHLH5* supports their roles as key nodes connecting JA signaling to diterpenoid biosynthetic gene expression. OsMYB133 is the first transcription factor identified to regulate both flavonoid and diterpenoid phytoalexin biosynthesis, directly activating *OsKSL4* and *OsCYP99A3* to promote sakuranetin and momilactone accumulation [23]. OsbHLH5 functions as a JA-inducible positive bifunctional regulator of phenolamide and diterpenoid biosynthesis, directly activating *OsCPS4* and *OsKSL4* [20]. This coordinated repression of a negative regulator and activation of positive transcriptional regulators collectively drives the metabolic output from JA signaling to phytoalexin accumulation.

A particularly notable finding is that the expression of diterpenoid biosynthetic genes and their upstream transcription factors was already elevated in OE plants under uninfested conditions, yet this did not result in elevated basal JA levels or visible phenotypic alterations. This pattern is diagnostic of a primed defense state—a configuration in which defense-related genes are poised for rapid induction but remain functionally restrained until an appropriate trigger is perceived [24,25]. Upon BPH infestation, the amplified JA burst in OE plants drives a further wave of transcriptional activation, leading to a robust and sustained accumulation of momilactones. This dual-layer mechanism—constitutive priming coupled with attack-triggered amplification—may explain how *OsJAC1* overexpression enhances resistance without imposing the growth yield trade-offs.

An intriguing aspect of our findings is the functional dichotomy of OsJAC1 as a positive regulator of BPH resistance, while a recent report using the same ZH11 background demonstrated that osjac1 knockout enhances resistance to rice blast and bacterial blight [11]. This apparent contradiction can be reconciled by considering the distinct defense signaling pathways activated by different classes of pathogens and pests. BPH is a piercing-sucking insect that typically activates JA-dependent defenses [26,27], which aligns with our observation that *OsJAC1* enhances JA accumulation. In contrast, the fungus *M. oryzae* and the bacterial pathogen *X. oryzae* pv. oryzae often trigger salicylic acid (SA)-dependent defenses while suppressing JA signaling [28]. *OsJAC1* may therefore act as a signaling node that differentially modulates defense outputs depending on the nature of the invading organism: amplifying JA-mediated responses against herbivores while restraining SA-mediated responses against certain pathogens. Such context-dependent functionality is not unprecedented for immune regulators in plants, as exemplified by the antagonistic cross-talk between JA and SA pathways [29]. We cannot exclude the possibility that a truncated OsJAC1 protein is produced in KO lines; however, the frameshift mutations are predicted to abolish protein function, and the KO lines exhibited increased BPH susceptibility, consistent with a loss-of-function phenotype. These observations underscore the complexity of *OsJAC1*’s role in plant immunity and highlight the need for careful consideration when deploying this gene in breeding programs.

From an applied perspective, *OsJAC1* represents a promising target for enhancing insect resistance in rice. Its overexpression confers significant BPH resistance without causing obvious yield penalties, and promotes the accumulation of diterpenoid phytoalexins under greenhouse conditions. However, the effect on field yield requires further investigation. Momilactone exhibits direct toxicity against chewing herbivores [30], and BPH feeding induces momilactone accumulation in rice leaves [31]. BPH honeydew-associated symbiotic microbes also elicit momilactone accumulation [32]. However, direct evidence for momilactone toxicity to BPH is currently lacking, and the link between OsJAC1-mediated momilactone accumulation and BPH resistance in our study is supported by genetic and correlative evidence. The primed defense mechanism established by OsJAC1 suggests that this gene could have broader applications in developing varieties with enhanced resilience against diverse biotic stresses. However, the context-dependent function of *OsJAC1*—acting as a resistance gene for insect defense but a susceptibility gene for disease resistance—must be carefully weighed in breeding applications. The identification of genetic backgrounds or environmental conditions that favor the beneficial effects while mitigating the trade-offs would be a critical next step. Additionally, exploring the upstream regulators of *OsJAC1* and its potential interactions with other defense signaling components may further illuminate the integration points among phytohormone pathways and could inform strategies for optimizing *OsJAC1*-mediated resistance in diverse agronomic contexts. Future studies should aim to test the direct effect of purified momilactones on BPH performance. However, although momilactone standards have recently become commercially available, they are supplied only in milligram-scale quantities at high cost, which is insufficient for the large amounts required for insect feeding assays. Heterologous expression of momilactone biosynthetic genes or chemical synthesis will be necessary to obtain sufficient pure compounds for artificial diet or membrane-feeding assays, which will ultimately establish causality and validate the role of these phytoalexins in rice–BPH interactions. While our genetic and correlative data support a model in which OsJAC1 modulates JA signaling and diterpenoid biosynthesis, the precise biochemical and molecular mechanism by which the OsJAC1 domains mediate these effects requires further experimental validation.

## 4. Materials and Methods

### 4.1. Plant Materials and Growth Conditions

The japonica rice variety Zhonghua 11 (ZH11) was used as the wild-type (WT) control. Using CRISPR-Cas9 technology, *OsJAC1* (LOC_Os03g01830) knockout mutants were produced by inserting an “A” at site No. 117 from “ATG” (*osjac1*-11) and a “T” at site No. 120 from “ATG” (*osjac1*-13) in the ZH11 background (Figure S1A), respectively. Agrobacterium-mediated transformation produced the *OsJAC1* overexpression rice lines (OE-23 and OE-25). Plants were grown in a greenhouse at 28 ± 2 °C, 70–80% relative humidity, with a 16 h light/8 h dark photoperiod. Two-week-old seedlings at the 4- to 5-leaf stage were used for all experiments.

### 4.2. BPH Infestation and MeJA Treatment

Brown planthoppers (*Nilaparvata lugens*) were maintained on susceptible rice seedlings (TN1) at 28 ± 2 °C, 70–80% relative humidity, and a 16 h light/8 h dark photoperiod. For BPH infestation, third-instar nymphs were placed onto rice plants at a density of 20 nymphs per plant for gene expression and hormone analyses, or 10 nymphs per plant for resistance assays. Leaf samples were collected at 0, 3, 6, 12, 24, and 48 h post-infestation for temporal expression analysis, at 0, 6, 12 and 24 h for hormone analysis, and at 0 and 24 h for transcriptome analysis. For MeJA induction, seedlings were sprayed with 100 µM MeJA (Sigma) containing 0.1% Tween-20; control plants received 0.1% Tween-20. Leaf samples were collected at 0, 3, 6, 12, 24, and 48 h after treatment. All samples were immediately frozen in liquid nitrogen and stored at −80 °C. Three biological replicates were performed per time point per genotype.

### 4.3. BPH Resistance Assays

Five uniform third-instar nymphs were starved for 1 h, weighed individually, and then enclosed in a Parafilm sachet attached to the basal stem of each rice plant. A pre-weighed piece of filter paper was placed at the bottom of the sachet to collect the excreted honeydew. After a 48-h feeding period under controlled conditions (26 ± 1 °C, 80% relative humidity, 14 h light/10 h dark), the filter paper was carefully removed, oven-dried at 60 °C for 30 min, and re-weighed using a microbalance to determine the honeydew weight. Concurrently, the surviving BPH nymphs were collected and re-weighed to calculate the individual weight increment. At least 15 insects per genotype were used for each assay, with three independent replicates.

### 4.4. Hormone Extraction and Quantification

Leaf tissues (150 mg) were harvested at 0, 12, and 24 h after BPH infestation, immediately frozen in liquid nitrogen, and stored at −80 °C. JA and JA-Ile were extracted and quantified using liquid chromatography-tandem mass spectrometry (LC-MS/MS) as previously described [33]. Phytohormones were quantified by comparing their peak area with the peak area of their respective internal standard as previously described [34]. Three biological replicates were performed for each time point.

### 4.5. RNA Extraction and qPCR Analysis

Total RNA was extracted from WT and OE-23 plants using TRIzol reagent (Invitrogen, Carlsbad, CA, USA) according to the manufacturer’s instructions. First-strand cDNA was synthesized using a PrimeScript RT reagent kit (Takara, Japan). Quantitative real-time PCR (qPCR) was performed using SYBR Green Master Mix (Takara) on a Real-Time PCR system (Bio-Rad, St. Louis, MO, USA). The rice Actin gene was used as an internal control, and relative expression was calculated using the 2^−ΔΔCt^ method. All primers used in this study are listed in Supplementary Table S1. Three technical replicates were performed for each biological sample.

### 4.6. Transcriptome Sequencing and Analysis

Total RNA was extracted from WT and OE-23 plants at 24 h after BPH infestation using TRIzon (Ultrapure RNA kit, CWBIO) according to the manufacturer’s instructions, with three biological replicates per genotype. Transcriptome sequencing was performed on the Illumina HiSeq X Ten platform at BGI Genomics Co., Ltd. (Shenzhen, China). After obtaining the raw data, low-quality reads were filtered out by SOAPnuke, and all clean reads were aligned to the rice reference genome sequence IRGSP-1.0 (NCBI RefSeq: GCF_001433935.1, MSU 7.0 annotation) [35]. For gene expression analysis, the read counts were normalized using FPKM (Fragments Per Kilobase per Million mapped fragments) method. Differential expression analysis was performed using DESeq2, with genes having |log_2_(fold change)| ≥ 1 and adj. *p* < 0.05 considered differentially expressed. The enrichment analysis of KEGG pathways was performed using the clusterProfiler R package, and enrichment with a *p*-value < 0.05 without multiple-test correction was regarded as significant.

### 4.7. Quantification of Momilactone A and Momilactone B

Momilactone A and momilactone B were extracted from leaf samples (approximately 200 mg) with or without BPH infestation for 24 h and quantified by LC-MS as in our previous work described [18]. Briefly, LC-MS analysis of rice diterpenoid phytoalexins (momilactone A and B) was conducted on an Agilent 1260 Q TOF MS system with an Extend C18 column under isocratic elution. Analytes were detected in positive ESI mode (*m*/*z* 250–400), identified by [M+H]^+^ exact mass, retention time and authentic standards and quantified using total peak areas. Three replicates were performed for each sample.

### 4.8. Statistical Analysis

All data are presented as mean ± SE from at least three independent biological replicates. Statistical significance was determined using Student’s *t*-test or one-way ANOVA followed by Tukey’s HSD test. Time-course qPCR data were analyzed by two-way repeated-measures ANOVA followed by a multiple comparisons test. A *p*-value < 0.05 was considered statistically significant. The statistical analysis was conducted using SPSS 20.0 (IBM SPSS, Somers, NY, USA), and graphs were generated using GraphPad Prism 7.0 (GraphPad Software Inc., San Diego, CA, USA).

## 5. Conclusions

In summary, we have identified OsJAC1 as a positive regulator that primes diterpenoid biosynthesis and enhances JA accumulation to enhance BPH resistance in rice. Transcriptomic analysis identified diterpenoid biosynthesis as the key pathway enriched in OE plants. qPCR and metabolomic analyses confirmed the activation of this pathway at both transcriptional and metabolic levels. Hormonal analysis revealed that OsJAC1 amplifies JA accumulation upon BPH infestation, driving the expression of transcription factors and downstream defense genes. This work provides new insights into the regulatory mechanism of rice defense against BPH and identifies *OsJAC1* as a potential target for enhancing insect resistance in rice breeding.

## Figures and Tables

**Table 1:** KEGG enrichment analysis of upregulated genes in OE vs. WT with BPH infestation.

Treatment	KEGG Pathway	ID	Up
WT VS. OE (CK 0 h)	Diterpenoid biosynthesis	osa00904	12
	Cutin, suberine and wax biosynthesis	osa00073	11
	Phenylpropanoid biosynthesis	osa00940	35
	Fatty acid biosynthesis	osa00061	9
	Amino sugar and nucleotide sugar metabolism	osa00520	18
	Fatty acid elongation	osa00062	7
WT VS. OE (BPH 24 h)	Phenylpropanoid biosynthesis	osa00940	9
	Diterpenoid biosynthesis	osa00904	15
	Cutin, suberine and wax biosynthesis	osa00073	1
	Ether lipid metabolism	osa00565	1

## Data Availability

The original contributions presented in this study are included in the article. Further inquiries can be directed to the corresponding authors.

## Supplementary Materials

The following supporting information can be downloaded at: https://www.mdpi.com/article/10.3390/plants15182840/s1. This transcriptome raw data has been deposited at https://www.ncbi.nlm.nih.gov/bioproject/?term=PRJNA1335611 (PRJNA1335611) accessed on 28 September 2025. Figure S1. Phenotypes of WT, OE and KO plants under normal growth conditions. (A) Sequencing results at target sites in the rice osjac1 mutant generated by CRISPR/Cas9-mediated gene editing in the ZH11 background. (B) *osjac1* mutants are more susceptible and *OsJAC1* overexpression plants are more resistant to *M. oryzae* compared to ZH11. Figure S2. PCA analysis of WT and OE samples at 0 and 24 h. Table S1. Primers used in this study. Table S2. List of differentially expressed genes (DEGs) identified by RNA-seq analysis (OE vs. WT at 0 h and 24 h).
