# Supplementary material for: OsJAC1 Positively Regulates Brown Planthopper Resistance in Rice Through Priming of Diterpenoid Biosynthesis and Amplification of Jasmonic Acid Signaling
Source: Plants (Basel). 2026 Sep 17;15(18):2840. doi: 10.3390/plants15182840 (PMC13610278; doi:10.3390/plants15182840)

Supplementary figures

Figure S1. Phenotypes of WT, OE and KO plants under normal growth conditions. (A) Sequencing results at target sites in the rice osjac1 mutant generated by CRISPR/Cas9-mediated gene editing in the ZH11 background. (B) osjac1 mutants are more susceptible and OsJAC1 overexpression plants are more resistant to M. oryzae compared to ZH11

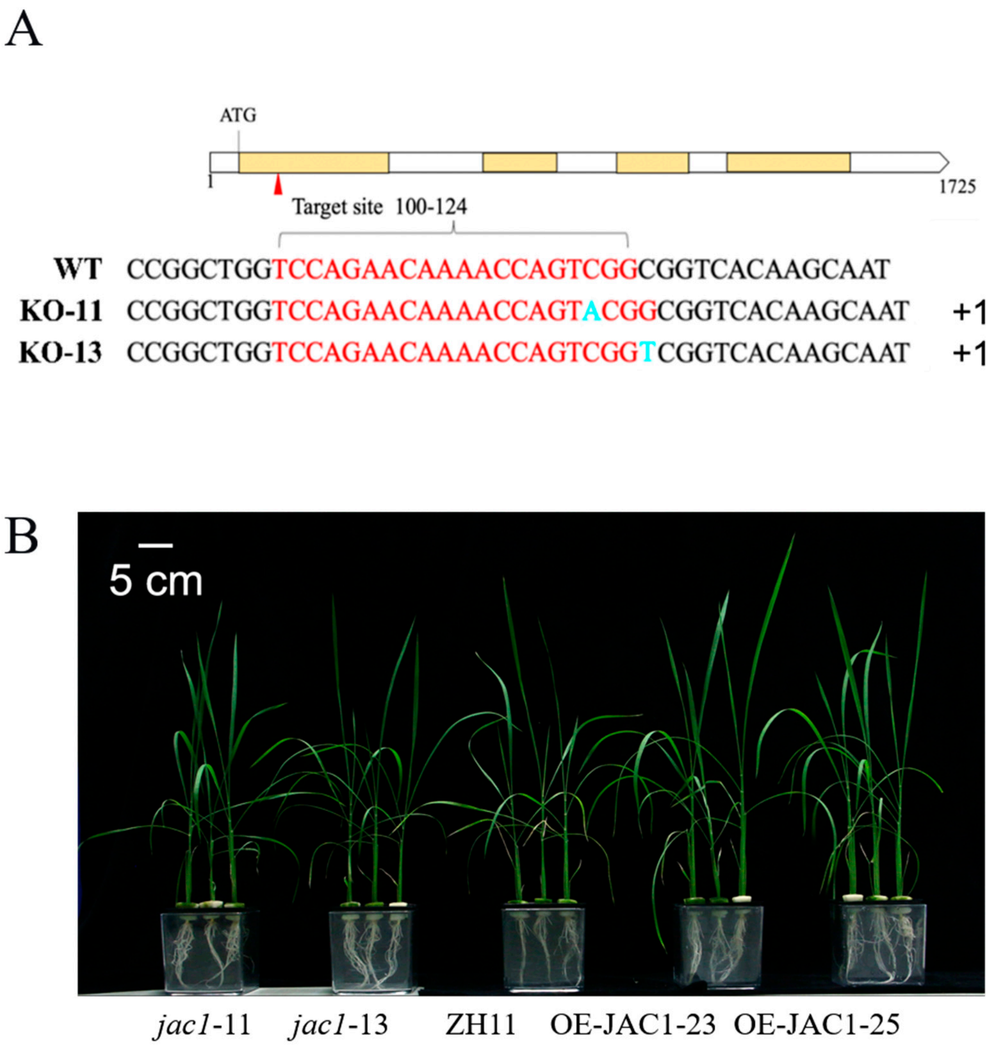

Figure S2. PCA analysis of WT and OE samples at 0 and 24 h.

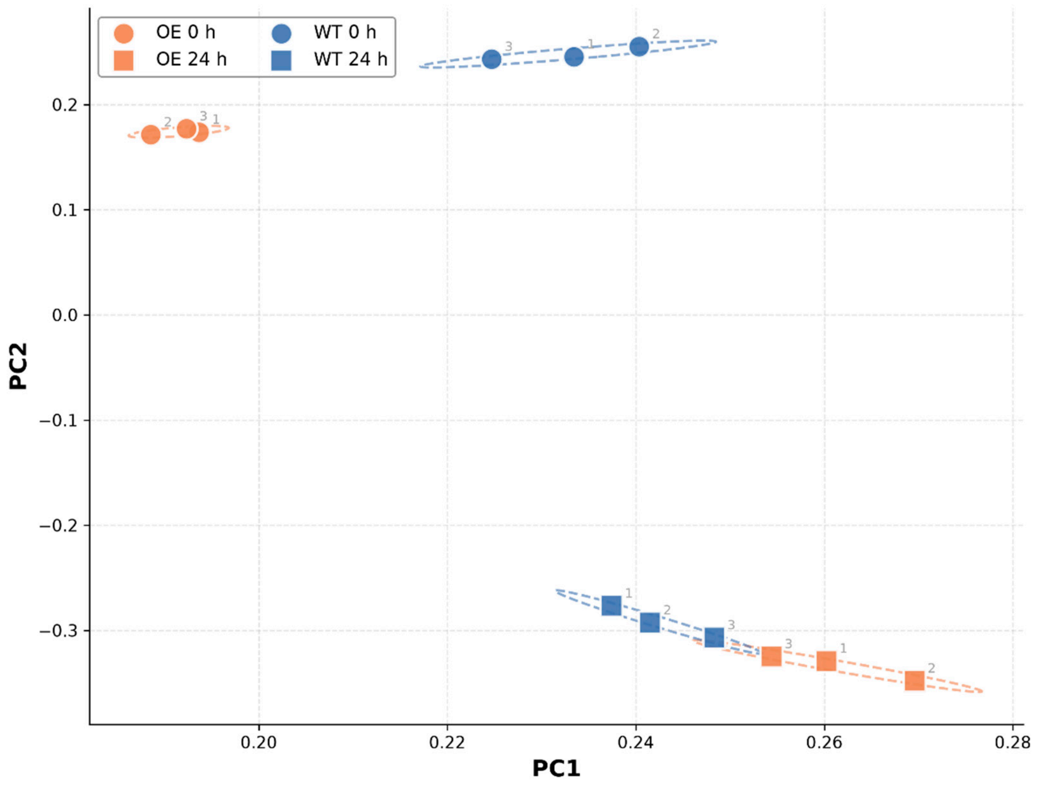

Supplement: Supplementary file 1 [file plants-15-02840-s001.zip › 00 Supplementary figures.pdf]
